# Perceptions of UK Community Pharmacists on Current Consultation Skills and Motivational Interviewing as a Consultation Approach: A Qualitative Study

**DOI:** 10.3390/pharmacy7020052

**Published:** 2019-05-31

**Authors:** Zahraa Jalal, Sania Akhtar, Katherine Finlay, Kathryn King, Neera Goel, Jonathan Ward

**Affiliations:** 1 School of Pharmacy, University of Birmingham, Birmingham B15 2TT, UK; akhtar.sy@gmail.com (S.A.); neera_goel@live.co.uk (N.G.); 2 School of Psychology and Wellbeing, University of Buckingham, Buckingham, Bucks MK18 1EG, UK; katherine.finlay@buckingham.ac.uk; 3 Florence Nightingale Faculty of Nursing, Midwifery & Palliative Care, King’s College London, London SE1 8WA, UK; kathryn.king@kcl.ac.uk; 4 Interactive Studies Unit, University of Birmingham, Birmingham B15 2TT, UK; J.D.Ward@bham.ac.uk

**Keywords:** community pharmacists, communication skills, consultation skills, motivational interviewing, behavioural consultations

## Abstract

**Objectives**: Community pharmacists’ roles in the UK are evolving; pharmacists currently deliver a wider range of clinical services with more patient-focused care. The objectives of this study were (i) to investigate UK community pharmacists’ views on their current communication skills in pharmacist-patient facing consultations, and (ii) to explore the perceptions of UK community pharmacists towards the application of motivational interviewing (MI) in a pharmacy consultation. In-depth qualitative face-to-face, semi-structured interviews with ten practicing community pharmacists were carried out, ranging from 30–60 min in length. The interviews were audio recorded, transcribed verbatim and thematic analysis was employed. Four themes emerged from the data: (1) the fight for time; (2) wrestling with consultation styles; (3) a personal communication evolution; and (4) unfamiliar but engaging motivational interviewing. These themes demonstrated the juxtaposition between the desire for patient-centred care and the pressures of managing broader dispensing work. Participants were critical of academic and continuous professional learning (CPD) training in communication skills and there was a strong recognition of the potential role of MI in promoting patient autonomy and outcomes. Participants recognized a few elements of MI techniques in their current consultations, but welcomed further training on behavioral change for effective consultations, expressing a desire for practical MI-specific training. Face-to-face CPD of consultation skills is needed to avoid the feeling of isolation among UK practicing pharmacists and rigidity in consultation delivery. Support for community pharmacists from other pharmacy staff could relieve current pressures and allow pharmacists time to develop and acquire effective skills for patient facing roles. Behavioural change consultation skills training for pharmacists could be an effective strategy to address these current challenges.

## 1. Introduction

Pharmacy plays a vital role in patient healthcare, often being the first port of call for many patients in the community, due to its ease of access [1]. For example, over 99% of the UK population are able to reach a pharmacy within 20 min walk [2,3] and on average 1.8 million people visit a pharmacy in the UK every day [2]. In addition, many pharmacies have long opening hours to meet patients’ and consumers’ needs [1]. This places pharmacies in an optimal position to deliver healthcare. Over the past four decades there has been a movement within pharmacy practice away from its original focus on dispensing, toward a focus on patient care [4]. The role of the pharmacist has advanced from that of a compounder and supplier of pharmaceutical products to that of a provider of clinical services [4]. This role is defined under pharmaceutical care as “the responsible provision of drug therapy for the purpose of achieving definite outcomes that improve a patient’s quality of life” [5] (p. 533).

UK community pharmacists offer public health services that target life style changes such as smoking cessation, weight management, emergency hormonal contraception, chlamydia screening, and advice on alcohol [6]. Pharmacists also provide reviews of the use of medicines in chronic conditions such as diabetes, obesity, high blood pressure and raised cholesterol [6]. They proactively promote a healthy living culture and liaise closely with local general practitioners (GPs), community-based nurses, health visitors and social care professionals. UK community pharmacists also deliver advanced clinical services; including medicine usage reviews (MURs), appliance usage reviews (AUR), flu vaccination service, NHS urgent medicine supply advance service (NUMSAS), stoma appliance customisation (SAC) and the new medicine service (NMS). Services such as MURs and NMS are designed to increase patient engagement with their condition and medicines and, support patients in decisions about their treatment to improve medication adherence and self-management [6]. The majority of pharmacy professional organisations advocate the potential of community pharmacists to extend their roles in patient care services. This is reflected in policy papers such as the “Five Year Forward View” for NHS England (2014) [7], the Nuffield Trust report Now More Than Ever (2014) [8] and The Future of Primary Care: Creating Teams for Tomorrow (2015) [9] which all call for a wider use of community pharmacists. The recently published NHS long term plan supports improvement in communication skills for primary care healthcare workers [10]. This is especially the case as the workforce is expanding to offer a greater range of treatment options for patients, including clinical pharmacists, advanced nurse practitioners, physiotherapists and mental health therapists and therefore shared decision making and personalised care become key components of communication skills [10].

A recent review on community pharmacy clinical services [6,11] called for the redesign of pharmacy services to make them a more integrated part of a multidisciplinary pathway of care. For example, evolving MURs into detailed clinical medication reviews with unlimited access to patient records whilst utilising independent prescribing [11]. Community pharmacy-based MURS have been found to reduce the risk of medication related problems and non-adherence and improve the appropriateness of medicine prescribing whilst producing substantial clinical benefits [12]. With such expansion in pharmacy roles, pharmacists need to focus on their communication and consultation skills to be able to deliver essential services [13]. Research shows that effective consultation skills can improve patient outcomes [14] and national guidelines on medicine adherence [15] indicate that healthcare professionals (HCP) should adapt their consultation style to the needs of individual patients and involve patients in decisions about their health [16]. This could be achieved by building a trusting relationship and rapport between the patient and the HCP [15]. A rise in patients with chronic conditions, reaching approximately 2.9 million in 2018 [17], average adherence rates for prescribed medications remaining at 50% and rates for lifestyle changes remaining below 10%, suggest that new approaches by HCPs may be required to influence patient behaviours.

The use of behavioural change strategies to increase self-care, decision-making and lifestyle change, are becoming increasingly common in healthcare settings [18,19]. Evidence from meta-analyses [20] support the use of behaviour change techniques such as Motivational Interviewing (MI) to improve medication adherence and clinical outcomes in patients with chronic diseases. This consultation technique has been introduced and has received increased attention in pharmacy communication skills education in the past decade [20,21,22]. More specifically, in the UK, the Centre for Pharmacy Postgraduate Education (CPPE) provides educational programmes which incorporate teaching on such consultation strategies, with scenarios and information on implementation [16]. MI refers to a counselling approach which is client-centred and is based on four key principles of expressing empathy, developing discrepancy, rolling with resistance and supporting self-efficacy [23,24,25]. MI is an “empathic, person-centred counselling approach that prepares people for change by helping them resolve ambivalence, enhance intrinsic motivation, and build confidence to change” [26] (p. 1).

Research has shown that MI can improve adherence to medication in different diseases such as chronic diseases, psychiatric disorders, HIV and asthma [20,27]. Evidence from clinical trials indicates that community pharmacists can deliver effective behavioural consultations, including MI, to improve medication adherence in a community pharmacy setting [28,29,30,31]. The evidence base for the successful inclusion of MI in pharmacy is growing; for example, a randomized controlled study including ten community pharmacies in USA [29] enrolled 216 patients with diabetes during a six-month period. Patients received MI pharmacy consultations as structured sessions incorporated in the pharmacy workflow with the aim to improve patient adherence to diabetes therapy. Patients in the intervention group showed a 6% increase in adherence compared with previous 180 days pre-intervention and a 28% increase in adherence compared with control group. Furthermore, in a large-scale community pharmacy study in Pennsylvania, USA [30] with 283 pharmacists, the intervention group were offered a brief screen that indicates a patient risk of non-adherence, followed by a pharmacist-led two-to-five-minute conversation using MI principles. The study included 29,042 patients with chronic diseases. Medication adherence for all classes of medication under study improved, ranging from a 4.8% difference in adherence for oral diabetes medicine to 3.1% for beta blockers. In addition, a UK study included 32 pharmacies from six London boroughs [31]. Intervention group community pharmacists delivered a 15–20-min MI consultation aimed at improving adherence to medication after a myocardial infarction. A statistically significant improvement in adherence was found at six months self-reported medication adherence among those receiving the intervention was 17% greater than that recorded in control patients [31].

Despite increasing evidence that MI can be beneficial to outcomes in the context of community pharmacy, there is currently no research addressing the concerns or knowledge base of the community pharmacists themselves. Surveying their opinions, perceptions and reports of pre-existing training in communication skills would significantly enhance the ability of future research to enhance the existing skillset of community-based pharmacists themselves and encourage increased uptake of CPD training courses in communication techniques. In this article, we explore UK community pharmacists’ views on their current patient consultations and on MI as a consultation approach for health-related behaviour change.

### Aims and Objectives

- To investigate UK community pharmacists’ views on their current communication skills in pharmacist-patient consultations.
- To explore the perceptions of UK community pharmacists towards the application of MI in a pharmacy consultation.

## 2. Materials and Methods 

### 2.1. Participants

In order to conduct the research with experienced pharmacy professionals, a homogenous, purposive sampling technique, as described by Etikan, Musa and Alkassin (2016) [32], was employed to achieve a relatively small sample (n = 10; 8 females, 2 males) of UK community pharmacists. Inclusion criteria were: practicing pharmacists, completion of a UK-based pharmacy degree programme, and current employment in pharmacies located in the West Midlands region. Community pharmacists not working in patient-facing roles were excluded from the study. Pharmacists were recruited through their managers whom had established links with the University (refer to Table 1). 

### 2.2. Procedure 

Participants were initially approached via email contact with Pharmacy Managers. Those pharmacists willing to take part were given an information sheet and interviews scheduled for a later date. After providing written, informed consent, semi-structured interviews were undertaken, ranging from 30–60 min in length. The interviews were conducted during a period of three months from beginning of November 2017 to the end of January 2018. The interviews were conducted face-to-face and were carried out in the pharmacy consultation rooms at the participants’ places of work by the researcher SA. All participants were debriefed after taking part and thanked for their participation. Literature regarding the attitudes of UK pharmacists towards communication/consultation skills, including MI, is limited, so an inductive approach was employed. 

### 2.3. Materials 

The interview discussion guide was semi-structured as sixteen questions and evolved around three main areas: (1) challenges, clinical pressures and consultation issues; (2) previous learning and CPD and; (3) MI: knowledge base, relevance and interest. A short, publically available video of a MI consultation was also shown as an exemplar to the pharmacists during the interview (see interview transcript in Supplementary Materials). The interview discussion guide was piloted with one pharmacist to ensure clarity and relevance of questions. The results from the pilot interview were not included in the results of this study. 

Ethical approval was obtained from the safety and ethics sub-committee of the University of Birmingham School of Pharmacy. 

### 2.4. Data Analysis

#### Thematic Data Analysis of Interview Transcripts 

Interviews were audio recorded, transcribed verbatim and thematic analysis was used to manually analyse the data, in accordance with the principles of Braun and Clarke (2008) [33]. Initial and repeated reading of full transcripts was carried out independently by two researchers SA and JW, to allow both to become familiar with the interviews in their entirety before any coding or identification of themes commenced. Recordings were also reviewed by SA in order to increase familiarisation with the data and to confirm accuracy of the transcripts. Once familiar with the breadth and depth of the data overall, the transcripts were independently analysed by the two researchers with data of interest highlighted in order to identify initial codes. This ‘blind coding’ approach was an attempt to ensure the integrity of the identified codes by reducing misinterpretation of codes, avoid the influence of pre-conceptions of individual researchers and reduce the possibility of individual researcher bias. An initial coding framework was developed as descriptive codes from individual transcripts and were grouped in clusters of related ideas, a process of constant comparison aimed to ensure that emergent themes were designated, reviewed and refined. Transcripts were initially analysed ideographically, before a cross-case analysis was undertaken, a third researcher ZJ, resolved any disagreements through discussion and close consideration of transcripts.

## 3. Results

Ten community pharmacists took part in the interviews. The sample included a mixture of both male and female professionals working within both independent and chain community pharmacies. Participants’ demographic data shown in Table 1.

### 3.1. Themes

Four superordinate themes emerged from the interviews: 1. The Fight for Time; 2. Wrestling with Consultation Styles; 3. A Personal Communication Evolution; and 4. Unfamiliar but Engaging MI (Please see Table 2).

#### 3.1.1. Theme 1—The Fight for Time

In community pharmacy practice, time pressures appeared to be the key concern in relation to consulting with patients. Pharmacists reported that dispensing activities placed pressure on clinical consultations and consultation length and time. For example, Pharmacist C commented:

“Most difficult is probably the pressure of time because there is rarely enough time within each consultation” Similarly, Pharmacist I suggested that: “Sometimes it’s the time factor where you feel like you’re pressurised to complete something within a certain amount of time”.

The majority of participants discussed the impact of carrying out patient consultations on other activities necessary for the effective running of their community pharmacies: “If you have more time in a consultation, then everything else in the pharmacy suffers” (Pharmacist D). Time spent with patients meant tasks reliant on the pharmacist built up, and this was experienced as an onslaught: “… the thing is, a lot of what I do is I have to go back into the dispensary to get injections and things if it’s a travel consultation, so as soon as you walk back into the dispensary you get bombarded” (Pharmacist J). As pharmacists’ own awareness of the likelihood of tasks building up in their absence grew, pressure increased and the fight for balance occurred:

“…there are lots of opportunities to do a lot of consultations and have a lot of communication with patients however, um, we have to balance that with carrying out all of the other jobs in the pharmacy that need doing that only a pharmacist can do”.

(Pharmacist B)

Seven of the pharmacists interviewed stated that time spent in a patient consultation was always an inherent concern and they felt frustrated by the fact that it constrained their ability to deliver both effective consultations and timely dispensing services: “Some MUR’s will take you minutes and then other ones, you’re sat in their ages and it is a bit annoying because you’re thinking, ‘Oh no, all of this is out there’”(Pharmacist E). The split between patient care and pharmacy management was experienced as an ongoing battle: “When you’re in the consultation room you’re needed outside as well as in the dispensary, so that is something that … you’re always living with … there’s always that pressure there” (Pharmacist J).

To cope with the balancing act and to fight against continual time pressures, the support of other pharmacy staff was deemed essential for effective consulting and some pharmacists had developed strategies for mitigating the impact of time spent with individual patients in consultation rooms:

“I’ve kind of got my staff trained so that if I’m absent they can still continue with everything else and get everything prepped out, they know to knock on the door if it’s urgent or at least explain to patients there might be a waiting time extended because pharmacist is busy with another patient”.

(Pharmacist A)

The team working made the fight for a balance achievable:

“… if you have a team that you can rely on and that you trust to get on with other things, to carry out whatever else needs doing in the pharmacy, then it’s a lot easier to comfortably sit down and speak to someone… you get to know one another and when you have that sort of trust relationship where you know what they’re capable of and you know what their competencies are you, can leave them in the pharmacy and get on with whatever you need to do”

(Pharmacist B)

However, commercial pressures, such as patients waiting in the pharmacy and specific targets to be met, remained even when support was perceived to be good. Workload pressures were highlighted as a reason for conducting brief consultations and the time available for effectively summarising and ending consultations was negatively impacted by dispensary duties: “I always found wrapping up is my worst thing… some patients will open up and talk to you about everything” (Pharmacist E). Increased demand from patients, insufficient time to do the job justice and workload pressure were all reported, whereas management support from other staff and colleagues were valued as a way to help relieve some of these pressures. 

#### 3.1.2. Theme 2—Wrestling with Consultation Styles

This theme reflected the personal and practice-based challenges of managing effective consultations that adhere to guidelines but can be responsive and flexible. Pharmacists recognised that consultations (as well as the role of pharmacists) may be changing and that current approaches to consultations may not be ideal for patients: “I think it tends to be probably very didactic, little bit dictatorial, offloading what you think might be good as opposed to listening to what the patient’s needs are, engaging with the patient” (Pharmacist C). The need to be flexible and responsive was recognised, but pharmacists felt that this was extremely difficult within the boundaries of the information they needed to gather and the traditional structure they were trained in:

“I think I’ve seen a lot of pharmacists go a bit rigid … because they have obviously to [keep] the consultation brief because of the nature of the work and so on, but they’re like ‘What medication are you taking? Can you tell me the medication you’re taking? Do you know what you’re taking it for? Did you ever forget to take a tablet?’ and I find this a very, very structured way of getting information”

(Pharmacist H)

Yet pharmacists demonstrated an awareness of the importance of building rapport and focusing on a patient holistically. They were also conscious of the role that listening effectively to patients takes in successfully building rapport:

“A checklist that you’re just ticking … it doesn’t have to have a certain chronological order. I think it’s the flexibility and the skills of gaining information without having to be going through a rigid schedule, that’s what helps you to build rapport because it feels natural”.

(Pharmacist H)

The majority of pharmacists felt that they could already build reasonable rapport with patients but recognised aspects of their consultation skills which could be improved; these included closing a consultation or further reflecting on a patient’s goal. 

Though the need for strong rapport was recognised, this conflicted with perceptions of more formalised pharmacy services. Pharmacy MURs and NMS are designed with a general structure and the value of this structure elicited contradictory views. To some degree, the structure was felt to be overly rigid, meaning that the natural flow of conversation was disrupted and difficult or sensitive topics neglected:

“I always think it [lifestyle issues e.g., weight loss, smoking] is something that’s thrown in at the end… I think if we were able to bring it in a bit more… and clearly having, ‘Yes you can have a chat with the pharmacist, it doesn’t have to be anything in particular’”.

(Pharmacist E)

To overcome this, some of the pharmacists stated that they did not commit to the structure and it was not used as a checklist: “So, if there were areas where the patient needed more information… I wouldn’t just stick to the guideline, I would use questions that are personalised to their health condition” (Pharmacist B). These pharmacists valued flexibility, reported that the structure could limit their focus on a patient’s concerns or needs and expressed a view that their ability to deliver a patient-centred consultation could be compromised by rigidly sticking to the consultation structure:

“I don’t know, I just guess I talk to them [as] more than just a patient, I kind of find out how they’re getting on, ‘How’s your day going?’, just talk to them as a human being rather than just a patient. If there’s anything they want to discuss we’re readily available, just so that they know they have someone to talk to”

(Pharmacist A)

Conversely, other pharmacists mentioned that they followed the structure as it helped keep the consultation more focused on the medication being dispensed and expressed the view that straying from the MURs/NMS guide would be more time-consuming and ineffective in achieving required results.

“… one advantage that you have is we use templates to help us structure our consultations, so the prompts are there and so the system itself will pick up whether they’re still smoking, they’ll pick up alcohol intake or elevated blood pressure or deranged bloods for some reason. So that in itself is a prompt, so particularly if you’ve got a patient who may be going off on a tangent and [there is] something clinically that you want to focus on, that will help”

(Pharmacist C)

The structure of the MURs/NMS was felt particularly to facilitate discussions that could be experienced as awkward: “I think MURs, it’s a little bit easier, because part of that criteria, like I said, was that we’d ask alcohol status, smoking status, counsel them on healthy eating, exercise and so on. So that’s a standard thing that we’re doing anyway, so actually that facilitates us to ask the questions that maybe we wouldn’t normally as a rule” (Pharmacist G).

However, as patients lacked knowledge about why/whether pharmacists would ask such questions in a standardised consultation structure, rapport could be jeopardised because trust and strength of relationship was not yet developed:

“…with a new patient because obviously they don’t know you, so you haven’t developed that rapport and that relationship. So introducing your role is very important introducing what you’re going to do through the consultation, how you can help them takes up a bit of that time, that initial time, whereas if you’ve seen patients previously or you’ve got a good working relationship with them, they’re clearer on how you interact with them or the benefits of your interactions as a pharmacist with them”.

(Pharmacist C)

Challenges caused by asking standardised questions were especially experienced in terms of sexual health:

“There are certain issues, like sensitive issues or sexual health, I find it most difficult to deal with especially when it’s the opposite sex asking questions along those lines”.

(Pharmacist F)

Pharmacists suggested that if patients were more aware of pharmacy public health services and encouraged to approach pharmacists for such services this would help patients be more open to discussions.

Pharmacists noted that working outside the boundaries of the MURs/NMS was beneficial and they reported often offering ongoing, brief support (such as checking progress informally when patients visit the pharmacy), perceiving of this as useful and effective:

“So existing patients, you have that rapport, you seem to know what would work best, whether they prefer quiet words or they’re happy to discuss things in open, would they prefer to talk to you directly, so that’s the thing with regular patients. New patients you have to try and show what you’re all about, so they have to trust you, trust your ability, so you’ve got to demonstrate that to them. You’ve got to prove yourself to them I think, so it takes a bit of time sometimes”.

(Pharmacist A)

The lack of an existing, established rapport with new patients meant that time had to be designated, within a potentially time-pressured consultation, for rapport building and to encourage a positive therapeutic, trusting relationship. Patients who were regular clients therefore removed the need for traditional consultation structure:

“… with existing patients you have that relationship with them and sometimes you’ve been through that whole journey of like from them starting their very first medication to now I don’t know being diagnosed with three or four conditions and being on lots of medication and when you’ve been through that relationship and you’ve got that rapport you’ve got that trust”.

(Pharmacist B)

Overall, pharmacists reported issues with current consultations and recognised the need to respect patient autonomy. They expressed a desire to treat patients as individuals, to understand patients’ perspectives/agendas and to run patient-centred consultations, echoing messages contained in numerous healthcare discipline guidelines and standards, and to have flexibility of approach depending on the patient. 

#### 3.1.3. Theme 3—A Personal Communication Evolution

The third theme demonstrated a recognition amongst participants that strong communication skills were only in their infancy during academic training, subsequently developing progressively in practice. Consultation skills training for the pharmacists interviewed were considered to have been limited at undergraduate level, and the time that had lapsed since initial academic training highlighted that teaching on communication skills was very variable. Some felt they had no specific interpersonal communication training: “For me more so because I did the degree a long time ago, it was only a three year degree, so it [communication skills] wasn’t covered then so it was pretty much from personal experience and CPD courses” (Pharmacist B). Whereas others recalled useful clinical skills:

“It’s been that long ago. Yeah, I wouldn’t say, from what I can remember, that I learned a lot on my undergraduate course with regards to consultation skills. There were sessions on it and some of the things that I can remember at that time I’m doing now, but a lot of the consultation skills I learned during my pre-reg and then first years as a pharmacist”.

(Pharmacist J)

Graduates that were more recent expressed that they had experienced slightly higher levels of consultation/communication skills training, but they were not targeted towards pharmacists:

“So we had, like, lectures on the models of communication but I don’t ever think they were necessarily the most helpful because they started off with doctors, and have been developed by doctors, so you have to pick and choose your favourite bits to bring it into pharmacy really; and I think we did quite a lot when we had dispensing classes for OSCEs and things, we had lecturers who would pretend to be on the phone”.

(Pharmacist A)

Even when such training existed and had been put into practice, it was mainly theory-based. Any role-play training described was limited to peer role-play or involved tutors playing the role of the patient (e.g., in telephone dispensing practise): “We did have some role play but I don’t think anyone took it seriously because you’re talking to a classmate” (Pharmacist I).

Due to the perceived limitations of academic training, consultation skills were felt to have been best learnt and developed during pre-registration or through early pharmacy career experience:

“Communication skills, it’s not an academic subject… it needs to be done through practice it’s not an academic subject, I think a bit of theory, whether it’s with your peers or with actors or with patients who are willing to help you and give you some feedback, I think that’s really important as well”.

(Pharmacist B)

Participants were consistently and bluntly negative about the academic training that they had received: “Um, I don’t know if I’ve learned anything from training sessions. I’ve learned a lot from my pre-registration tutor” (Pharmacist E). Though CPD is intended to promote ongoing learning and communication-based CPD is available, the predominantly online nature of CPD provisions was felt to be inadequate:

“I think we can do CPD but there’s nothing compares to having face to face training. That’s what I think. So, yeah, we can do online based CPD or even where we have a CPD event and they’re OK, but I think there’s nothing like face to face training where you’re getting feedback, so that may be peer to peer, it may be in a university setting or like this or just with patients and getting feedback from patients”.

(Pharmacist F)

Participants valued practical learning and found that the potentially isolated nature of pharmacy work plus over-reliance on online CPD could lead to social and professional isolation. This was specifically felt to be due to a lack of opportunity to observe others in comparable contexts:

“I never listen to anybody else do a consultation, other than perhaps in the CPPE manual on the YouTube thing, so I don’t really know what everybody else will do because I don’t work with another pharmacist”.

(Pharmacist J)

Within this theme there was a universally negative perception of current training and CPD provisions in relation to inter-personal communication, yet all participants displayed a significant desire for personal development and growth in practice. Community pharmacists CPD face to face learning of consultation skills could help support development.

#### 3.1.4. Theme 4—Unfamiliar but Engaging MI

The final theme reflected both a lack of knowledge about Motivational Interviewing (only one interviewee had heard of motivational interviewing), alongside a rapid engagement and interest in MI’s potential once participants were exposed to information about the methods. Once demonstrated, using a publicly available video of a MI consultation, it quickly became apparent that the majority of the interviewed pharmacists recognised where it could enhance patient-centred communication: “I like it [MI] very much actually because it’s more about the patient, rather than bombarding the patient with information” (Pharmacist J). In addition, participants felt that they were already implementing some aspects of MI in their practice: “Yeah I think it’s really good [MI] and like you say you do do it even if you didn’t know what it’s called” (Pharmacist E). For example, many respondents recognised the use of open-ended questions during their consultations: “I’ll use an open question saying, ‘What do you think about your current weight?’” (Pharmacist C). Some common MI techniques, such as the use of summaries and scales, however, were unfamiliar. These were not considered standard pharmacy approaches and participants found their use engaging:

“Yeah, summarising, is that what it was? I probably am not the best at that - I’ll probably just tell them the once and the whole scale thing I thought that was really good because he went from like a 7 to a 10 and that was really good because he thought oh yeah there’s some issues here isn’t there and then he said yeah I’m a 10 now all of a sudden”.

(Pharmacist C)

Overall, participants were quick to observe and comment positively on MI skills and to note their potential utility.

Even with their minimal knowledge of MI approaches, pharmacists reported that they recognised the importance of empowering the patient to manage their own health and promote autonomy by personalising care: “I think it’s a lot easier to give the patient something that they can actually achieve rather than just, you know, generalising them - I just think it’s a lot easier to give them their own sort of goals ”(Pharmacist C). Participants expressed a curiosity about whether MI could imbue their own practice with more confidence and help them to achieve better clinical outcomes: “With more challenging patients, [it would] help to develop confidence and help them to address more health concerns so that you’ve got, not just patient outcomes, a more holistic approach to care” (Pharmacist B). As a consequence, pharmacists were quick to request further training on conducting consultations and 9/10 of the pharmacists interviewed felt that effective training on MI would support them in their enhanced clinical role: “I think it [MI training] would be interesting, and interesting to look at what I already do that comes under that bracket and how I can then improve” (Pharmacist E). This interest was in terms of a desire for practical training which could have personal benefits alongside facilitating more effective consultations for patients: “I think it [MI] would make such a massive difference in consultation skills. At the moment I think a lot of pharmacists are apprehensive in doing consultation skills just because there is a lot more emphasis holistically about patients now rather than just their medication” (Pharmacist A). The results of the interviews clearly demonstrated a desire for MI-specific hands-on training:

“I am very interested, I’d like to find out if there’s any courses being run, if they are, are they nation-wide or are they isolated, are they pilot schemes, has this actually been introduced anywhere and if it has, has there been any difference. Probably trial it out on some patients themselves”.

(Pharmacist F)

## 4. Discussion

### 4.1. The Fight for Time

Pharmacists in this study reported struggling with dispensary pressures affecting the time which could be devoted to pharmacy service consultations which, in turn, had an effect on the breadth and length of current patient consultations. Similarly, pharmacists’ time and high dispensary work load as a barrier to effective pharmacy consultation has been highlighted in previous studies investigating feasibility of delivering clinical services at community pharmacy level [34,35,36]. Consultation time was reported by participants in this study as having an impact on daily tasks, with pharmacists feeling that they had to rush consultations when aware that the workload of dispensing was building up. This finding is in line with a UK focus group study of 22 community pharmacists [37] which found that community pharmacists reported they had less time to counsel patients than they would like. A previous UK qualitative study [38] including observations of MURs in community pharmacies showed that UK community pharmacists’ heavy commitment to the dispensing process meant there was poor integration of clinical services such as MURs into routine workload. Therefore, policy and structure changes in community pharmacy practice need to occur to allow pharmacists time to incorporate clinical services within their daily routine [38]. This could also be achieved if proper support from other pharmacy staff is available. Pharmacy technicians are registered professionals working in a similar pharmacy environment as pharmacists and can potentially release pharmacists’ time to undertake clinical responsibilities. This has had support of professional bodies and is reported in policy and discussion papers such as General Pharmaceutical Council’s (GPhC – UK regulator for pharmacists) ‘Tomorrow’s pharmacy team’ [39] that highlights the important role of pharmacy technicians, who could help support many of the services aiming to extend the clinical role of community pharmacy. 

### 4.2. Wrestling with Consultation Styles

In the current study, pharmacists reported that they were satisfied with the rapport that they currently build with their patients. In the UK the GPhC in 2017 produced nine new standards for pharmacy professionals that describe safe and effective care delivered through ‘person-centred’ professionalism. These standards reflect what people expect from pharmacy professionals [40]. Among these nine standards are to communicate effectively. In this standard of care “communication” means to ensure that effective care is delivered, pharmacists need to adapt their communication skills to meet the needs of the person. In addition, to be able to build rapport pharmacists should come to a shared decision with their patients [40]. In healthcare, trust is important to provide better care [41]. A survey report published in 2015 by the GPhC [42] showed that in a sample of 1,160 members of the general public, almost 9/10 respondents (87%) said they trust health advice from a pharmacist. This was a similar finding to trusting advice from other allied HCPs but lower than GPs (95%). Therefore, rapport could be achieved if trust existed between the patient and pharmacist.

In this study, different views were found among the interviewed pharmacists regarding the use of consultation structured guides for MURs and NMS consultations. Where some pharmacists followed a rigid structure, others preferred a flexible consultation approach. Both the MUR and NMS consultation guides contain an interview schedule with a series of open-ended questions to facilitate the consultation and should be adapted accordingly to each patient consultation. Despite this flexibility some pharmacists in this study reported preference to follow a rigid checklist to both focus and save time on a consultation. UK educational bodies that provide training packages that fulfil the service specification for such consultations (Centre for Pharmacy Education in collaboration with other pharmacy UK bodies, for example Pharmaceutical Services Negotiating Committee PSNC and NHS Employers) state that these NMS and MURs guides are carefully developed with academic input from experts in pharmacy and psychology and that there is no absolute requirement to use the questions in a rigid manner [43]. Furthermore, a previous UK study showed that [38] when examining counselling practices in MUR consultations, MURs where found to be brief, dominated by closed questions and that pharmacists adhered to its ‘tick-box’ format. This enabled the MUR form to be completed efficiently, but affected opening a wider discussion regarding the patient’s health [38]. A large UK observational comparative study investigating community pharmacists’ current practice while using these interview schedules during pharmacy consultations could be of benefit. This could help determine which approach (flexible versus rigid) could be more beneficial when trying to incorporate patient-centred care into a pharmacy consultation.

### 4.3. A Personal Communication Evolution

Pharmacists’ satisfaction with previous training on consultation skills at undergraduate level was low in this study and pharmacists reported that they gained and developed their skills mostly during practice pre-registration period and after graduation. Other UK studies have reported similar findings; in a national survey including 700 UK pharmacists, 271 participants evaluated their consultation skills at undergraduate level and how equipped they felt to be able to conduct patient consultations [37]. On a scale where one was not prepared and five was fully prepared, the mean rating was 3 with over half of the participants developing their consultation skills after registration and the majority of pharmacists expressing a need for further training. A recent UK survey of 109 practicing community pharmacists by the authors of this paper [44] also showed that more than half of the participants in the survey who were taught consultation skills at an undergraduate level reported that they were not satisfied with their undergraduate training; ranking their consultation skills training as below 5 on a scale of 1 to 10 with 10 been very satisfied. 

Perhaps pharmacy educators can draw on the significant body of literature available in relation to the development of clinical communication curricula of other clinicians such as nurses, doctors and psychologists. For example, within UK medical schools since the 1980s, surveys of communication skills teaching at UK medical schools [45,46,47] offer insights into methods of teaching and assessment of communication skills. More recently, the UK Council of Clinical Communication in Undergraduate Medical Education (UKCCC) released an updated version of its diagrammatic ‘communication curriculum wheel’ and accompanying consensus statement which recommends key domains, tasks and skills to be included in undergraduate communication curricula [48]. In their commentary on the original UKCCC model, Kinnersley and Spencer (2008, p. 1053) [49] note that, “Although crafted with UK medical schools in mind, the model may be transferable to curricula for other health professionals” such as pharmacists. 

The feeling of isolation from other pharmacy colleagues and the ability to reflect on each other’s consultation skills was also evident from the interviews, which could be due to the nature and structure of community pharmacy practice in the UK. Research investigating effective interventions to improve communication between consumers and pharmacy personnel during OTC consultations in the community pharmacy setting, found that active learning techniques such as face-to-face training with role-play could potentially improve pharmacists’ communication skills whilst minimising feelings of isolation [50]. Therefore, CPD training opportunities should allow practicing pharmacists an opportunity to observe other colleague’s consultations and allow peer review.

### 4.4. Unfamiliar but Engaging MI

Interviewed pharmacists related certain aspects of their consultations with some principal components of MI and welcomed further training opportunities regarding behavioural change approaches. While some aspects of consultations might be familiar such as the use of open-ended questions, however, MI differs significantly from traditional methods pharmacists use to communicate with patients and requires training and practice [25]. MI is unlike traditional counselling techniques that commonly include provision of information and education by a HCP who is usually an expert, expects respect and dictates a healthcare behaviour, to motivate the patient towards change [25]. While in motivational interviewing a partnership is developed between the patient and HCP, to reach an informed decision; it is based on sharing, exchanging information and negotiating behaviour to reach an agreement for change, a HCP in this consultation will need to earn respect and accepts patient’s behavior [25]. MI is a patient focused and centred consultation [25]. It could be therefore, useful to add components of behavioural change techniques within an MUR/NMS structured guide.

Finally, recent moves towards large dispensing factories, internet pharmacies, ‘Hub-and-spoke’ dispensing [51] and robotics may not currently be universally welcomed; but these together with trained accuracy checking technicians could potentially free up community pharmacists’ time to provide clinical services, person-centred consultations and relieve dispensing workload pressures [36]. Furthermore, effective communication skills including MI and behaviour change technique could all act to support the NHS long term plan [10]. 

### 4.5. Limitations

Limitations of this study included a small sample size of 10 participants; however, themes identified and messages taken from the limited number of interviews were broadly consistent. Participants’ workload and availability had an effect on the length and number of interviews, however, such pressures are UK-wide. The majority of the participants were permanent staff. Only three of the participants were locum pharmacists, which could have led to different perspectives especially in areas of building rapport with patients. Pharmacists interviewed all based in the West Midlands area, which could affect generalizability. 

### 4.6. Implications for Practice and Policy

There is a need for pharmacy educators to focus on development and assessment of teaching of communication/consultation skills at undergraduate level, in order to be able to provide pharmacy graduates with appropriate training to meet their clinical roles. Furthermore, qualified pharmacists are in need of structured and standardised advanced CPD training to be able to provide patient-centred consultations and optimal patient care. Motivational Interviewing should be considered a strong candidate for the development of practical CPD training.

## 5. Conclusions

Community pharmacists expressed an urgent need for further development of their consultation skills. In line with this, pharmacists need to have sufficient time when dealing with patients directly. Training pharmacists on behavioural change consultation techniques could be the way forward to shift care to a patient-centred approach.

## Figures and Tables

**Table 1:** Participant Demographics.

Participant ID	Age Range	Gender	Highest Education Level Achieved	Years of Practice	Pharmacy Sector
A	20–30	F	Undergraduate	1–5	Community
B	40–50	F	Undergraduate and IP*	16–25	Community/primary care
G	30–40	F	Undergraduate	6–10	Community
F	30–40	M	Undergraduate and IP	11–15	Community
E	20–30	F	Undergraduate	1 year	Community
I	20–30	F	Undergraduate	1 year	Community
J	40–50	F	Postgraduate	11–15	Community
C	20–30	M	Undergraduate	1–5	Community
H	30–40	F	Postgraduate	6–10	Community
D	30–40	F	Undergraduate and IP	6–10	Community

* IP-Independent prescribing course.

**Table 2:** Identification of themes.

Superordinate Themes	Codes Identified
Theme 1 The Fight for Time	Dispensing & time pressures - Waiting customers - Targets Activities other staff cannot perform Existing patients old versus new Time limited consultations
Theme 2 Wrestling with Consultation Styles	Building rapport Patient-centred consulting Following a consultation structure Flexibility of approach required Patient autonomy Difficult/sensitive issues in consultations
Theme 3 A Personal Communication Evolution	Limited undergraduate training Online training only (CPD/postgraduate) Focus on theory based training & associated limitations Lack of professional simulated patients (SPs) in role play training
Theme 4 Unfamiliar but Engaging MI	Lack of awareness of MI (and knowledge base) Positive response to MI concept - Potential for improved patient outcomes - Improvements in confidence of pharmacists Interest in MI training Open questions Summarising Patient-focused consulting, Patient autonomy

## Supplementary Materials

The following are available online at https://www.mdpi.com/2226-4787/7/2/52/s1, Table S1: Interview Transcript.
