# Supplementary material for: Perceptions of UK Community Pharmacists on Current Consultation Skills and Motivational Interviewing as a Consultation Approach: A Qualitative Study
Source: Pharmacy (Basel). 2019 May 31;7(2):52. doi: 10.3390/pharmacy7020052 (PMC6630739; doi:10.3390/pharmacy7020052)
Supplement: Supplementary file 1 [file pharmacy-07-00052-s001.pdf]

Interview Transcript:

1) Seeing patients in your pharmacy involves the use of many different communication and consultation skills; can you tell us about what you find easiest/most difficult in terms of communicating with patients?

Prompts: - How are consultations with new/existing patients different? - How do you balance listening to patients with clinical and commercial time pressures? - How do you usually manage your consultation regarding time? - How do pharmacy staff help with dispensing duties when you are occupied with a pharmacy consultation?

2) Encouraging patients to make a positive health-related behaviour change requires good relations with the patient; explain how you build rapport with patients?

Prompts: - What would you most want to change about current consultations? - What kinds of difficulties do you have with current consultations? - How regularly do you expect to counsel the same patient?

3) Pharmacies offer services to help patients lead a healthier lifestyle. How do you try to gain information about the patient's lifestyle and choices in their health related behaviours?

Prompts: - How do you tailor the consultation towards the specific patient's health related behaviour change? - Explain how current consultations make this easy or difficult?

4) How are you able to explore the reasons behind the patients desire or reluctance to change their behaviour?

Prompts: - Explain how you think current consultations encourage patients to regularly revisit the pharmacist, regarding health related behaviour changes? - Currently, how are you able to recognize when a patient is ready to make a health-related behaviour change?

5) How are your current pharmacy consultations structured?

Prompts: - Explain how you would conduct an MUR/NMS?

6) After having experience in consultations with patients, can you explain how you think pharmacists may benefit from further training in consultation and communication skills?

Prompts: - How long do you feel a consultation with a patient should be and why? - Do you think this is being achieved with current consultations? - If applicable, how do you apply/practise new skills that you learn from training sessions?

7) Describe any postgraduate courses you have undertaken in order to further develop your consultation skills? E.g. Online courses, workshops, face to face sessions.

Prompts: - What did you learn from these courses? - How did these courses add to your knowledge of current communication skills?

8) How satisfied or dissatisfied were you with your undergraduate course/courses on consultation skills with patients?

Prompts: - Who would deliver the course? - How was the course delivered? - How long was the course delivered for?

9) How familiar are you with the term motivational interviewing? - 'I have heard of it' - Can you please tell me what you know about it already? - Have you ever been trained on motivational interviewing? - Have you read any papers/journal articles on MI or have you been part of research involving MI? - 'I have not heard of it' - Motivational interviewing is a term used for a counselling approach developed by two clinical psychologists, William Miller and Stephen Rollnick. It focuses on engaging with the patient in order to motivate them to change their own behaviour. The main way of doing so is by encouraging patients to explore the reasons as to why they may be reluctant to change their behaviour and by coming up with a plan in order to motivate them to achieve their goal. It differs from traditional MUR counselling approaches in which the pharmacist explains the benefits of health-related changes and make suggestions. In motivational interviewing, however, the pharmacist will actively explore the reasons behind the patient's choices and together, they will come up with a plan to achieve the goal they have set together. The strategy helps patients to reflect on their behaviour and consider how a change in behaviour may benefit them.

HealthTeamWorks, coloradoguidelines. Motivational Interviewing: Evoking Commitment to Change 2009. [cited 29 April 2019]. Available from: https://www.youtube.com/watch?v=dm-rJJPCuTE

Upon hearing about this, how would you define motivational interviewing if you were trying to explain to someone else? - Consultation skills for pharmacists taught at university may or may not share similarities to Motivational Interviewing, can you describe any similarities/differences you may recognise after watching the video?

10) Now that you have an idea of what it is, what are your first impressions?

Prompts: - Explain if any obstacles you face currently in terms of communication with patients will be overcome by implementing motivational interviewing as a consultation approach?

11) Have you ever learnt anything similar at university? - Yes - when did you qualify?

12) Can you please describe what you expect a patient-centred consultation to be? - What key verbal and non-verbal skills do you think are required for pharmacists? - What other popular models of consultations have you heard of? E.g. Health Coaching?

13) Motivational Interviewing relies on several different consultation skills, such as the ability to ask open ended questions, the ability to provide affirmations (relaying to the patient that you understand their point), the capacity for reflective listening and the ability to periodically provide summary statements about the consultation to the client. - Can you please tell me more about how you think such skills may influence your consultation with patients?

14) How do you think pharmacists, and therefore patients would benefit from training in Motivational Interviewing?

Prompts: - Is there anything you recommend? 62 - How do you think patients could benefit more from current consultations?

15) Do you think motivational interviewing or current consultations will benefit patients more? - Motivational Interviewing – why? - Current – why?

16) After briefly learning about motivational interviewing as a consultation approach, can you explain your thoughts on how you think implementing motivation interviewing might affect your consultation?
